# Supplementary material for: Young KRAB-zinc finger gene clusters are highly dynamic incubators of ERV-driven genetic heterogeneity in mice
Source: Nat Commun. 2025 Oct 30;16:9608. doi: 10.1038/s41467-025-64609-2 (PMC12575710; doi:10.1038/s41467-025-64609-2)
Supplement: Supplementary file 1 — Supplementary Information [file 41467_2025_64609_MOESM1_ESM.pdf]

Supplementary Information

Supplementary Data file description

Supplementary Data 1
Curated annotation of KZFP genes in the Chr4 cluster in BL6J, 129S1 and CAST mouse strains.

Supplementary Data 2
Zinc fingerprint arrays of coding KZFP genes in the Chr4 cluster in BL6J, 129S1 and CAST mouse strains.

Supplementary Data 3
TE content and enrichment at examined KZFP gene clusters in rat, *Mus pahari*, *Mus spretus*, *Mus musculus* (BL6J, 129S1 and CAST strains) and *Homo sapiens.*

Supplementary Data 4
Percentage of divergence of LTR elements in BL6J, 129S1 and CAST strains, and *Mus spretus*.

Supplementary Data 5
Enrichment analysis of BL6J Chr4cl KZFP ChIP-seq peaks over TEs.

Supplementary Data 6
Summary of BL6J Chr4cl KZFPs' binding preferences: target TEs and sequence motifs.

Supplementary Fig. 1

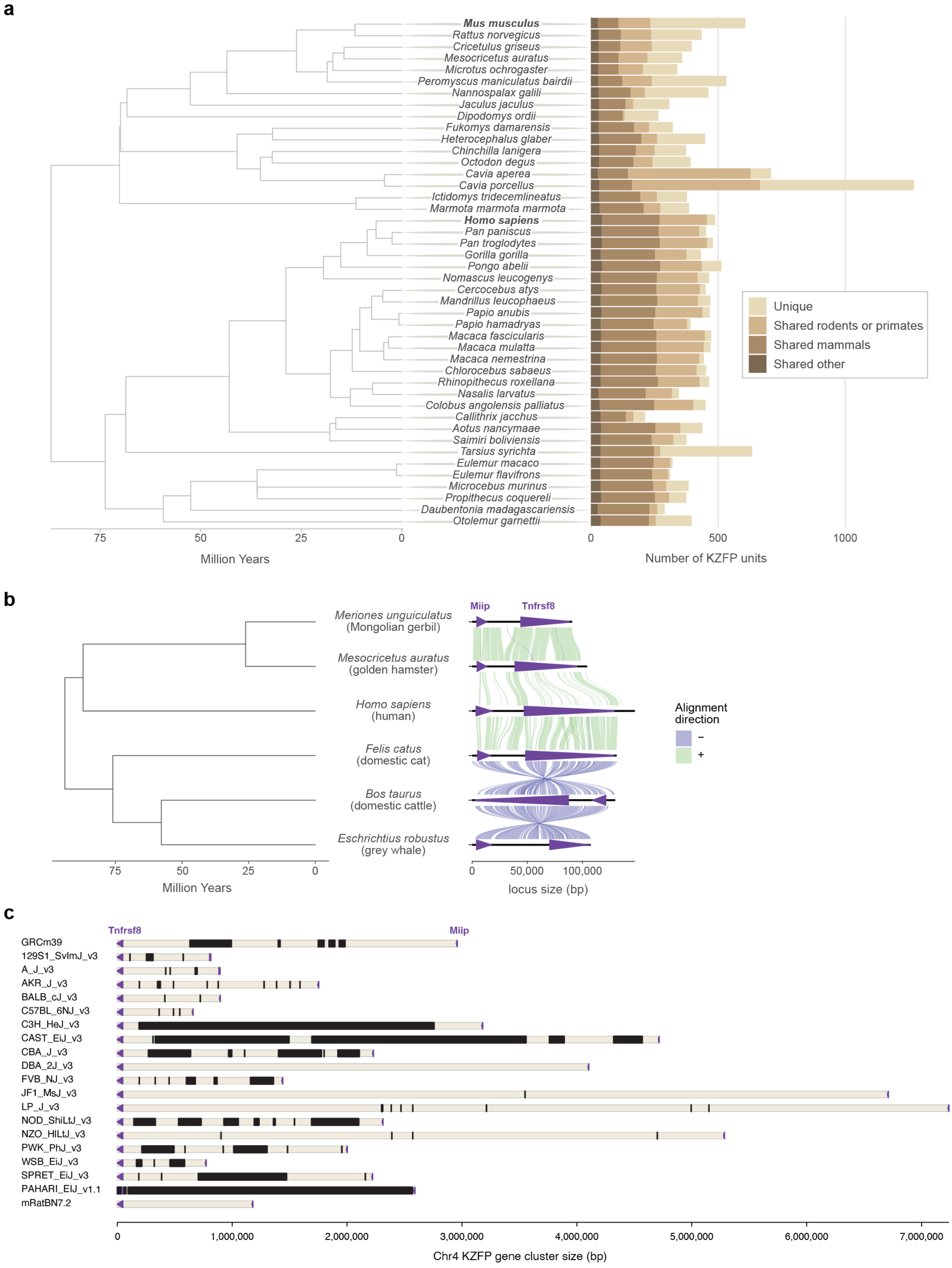

Supplementary Figure 1. Mice display several species specific KZFP genes and KZFP gene clusters still retaining sequence gaps.

a, Number of KZFP units in all rodent and primate species analyzed in Imbeault et al. 2017 [1]. From lighter to darker color: KZFP units unique to the species (compared to all species analyzed in Imbeault et al. 2017); KZFP units shared with at least one other rodent (for rodents) or primate (for primates) species; KZFP units shared with at least one other mammalian species besides rodents or primates; KZFP units shared with at least one other species besides mammals. **b,** Conservation of the *Tnfrsf8*-*Miip* gene block around the mouse Chr4 KZFP gene cluster locus across the mammalian clade, by comparing the locus in Mongolian gerbil (Bangor_MerUng_6.1), golden hamster (BCM_Maur_2.0), human (GRCh38.p14), domestic cat (F.catus_Fca126_mat1.0), domestic cattle (ARS-UCD2.0) and grey whale (mEscRob2.pri). **c,** Annotation of sequence gaps at the Chr4 KZFP gene cluster locus in the most recent reference assemblies for 16 mouse strains, with the addition of *Mus spretus*, *Mus pahari* and rat (RN7.2 assembly). Importantly, most gaps have been annotated as of unknown size. Gaps are indicated in black; *Tnfrsf8* and *Miip* genes are displayed as purple arrow heads.

Supplementary Fig. 2

a

	assembly general stats				BUSCO (eukaryota)					BUSCO (glires)				
**Assembly**	**total bp**	**contig number**	**contigs N50**	**contigs aligned to mm39**	**Complete (all)**	**Complete (single-copy)**	**Complete (duplicated)**	**Fragmented**	**Missing**	**Complete (all)**	**Complete (single-copy)**	**Complete (duplicated)**	**Fragmented**	**Missing**
**BL6J (all contigs)**	2993385643	1477	100Mb	211	99.60%	92.90%	6.70%	0.40%	0.00%	96.40%	93.50%	2.90%	0.60%	3.00%
**129S1 (all contigs)**	2907737724	2548	109Mb	172	99.70%	92.20%	7.50%	0.40%	0.10%	96.40%	93.50%	2.90%	0.60%	3.00%
**BL6J (filtered contigs)**	2636461246	38	101Mb	38	99.60%	92.90%	6.70%	0.40%	0.00%	96.30%	93.40%	2.90%	0.50%	3.20%
**129S1 (filtered contigs)**	2613050807	37	120Mb	37	99.70%	92.20%	7.50%	0.40%	0.10%	96.50%	93.60%	2.90%	0.50%	3.00%

b

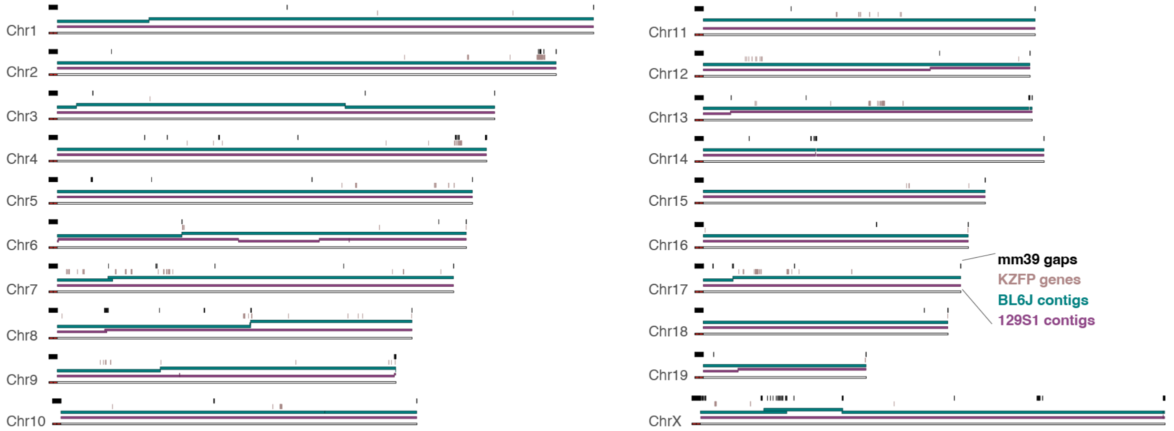

c

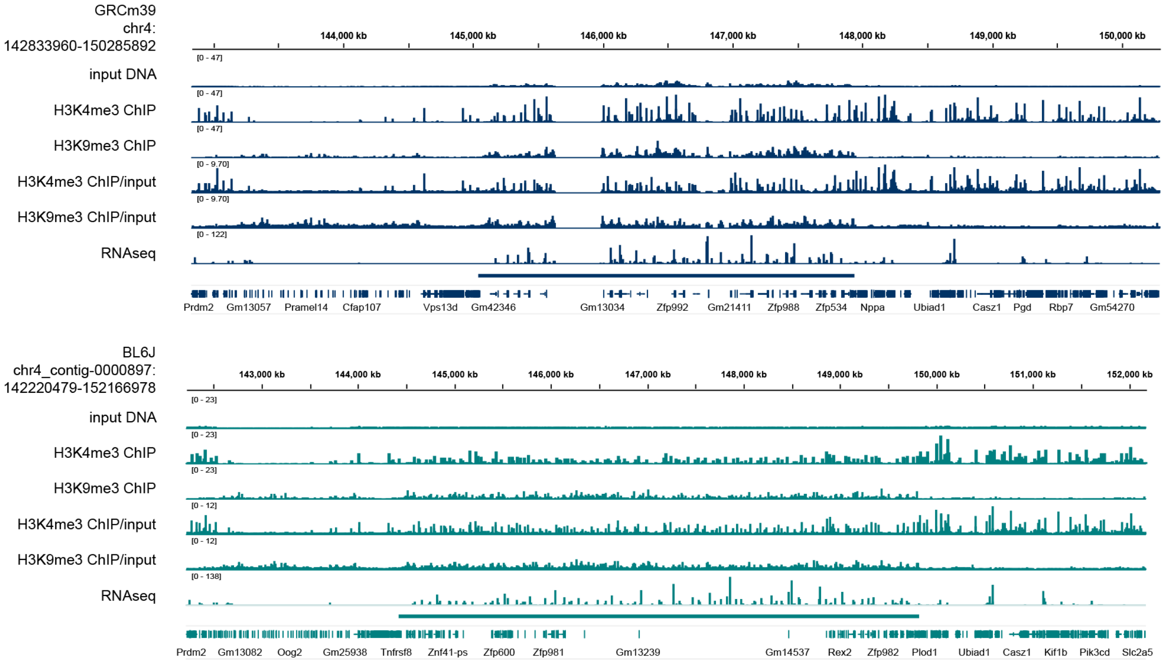

d

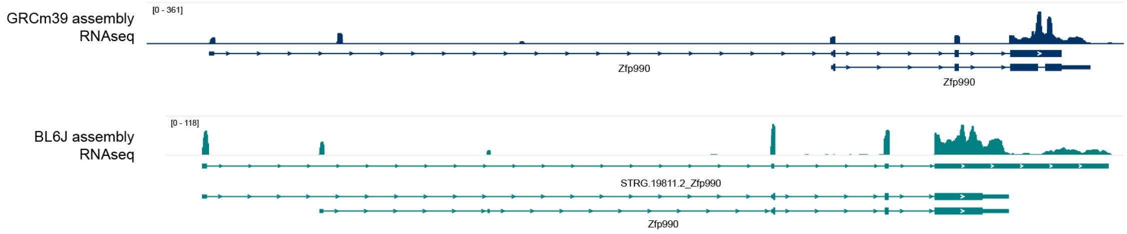

Supplementary Figure 2. Gapless assemblies of KZFP gene clusters prevent artificial read pileups.

a, Statistics of BL6J and 129S1 de novo assemblies from this study, before and after contig filtering and strand correction. **b,** Ideogram based on the GRCm39 assembly, displaying the alignment spans of contigs retained after filtering and strand correction in both the BL6J and 129S1 de novo assemblies. BL6J contigs are indicated in teal, 129S1 are indicated in magenta. **c,** IGV snapshots of H3K4me3

and H3K9me3 ChIP-seq experiments performed in pure BL6J strain mESCs (HGTC8 cells), as well as RNA-seq data (published dataset [25]) at the Chr4 KZFP gene cluster locus. Reads were mapped to either the GRCm39 reference assembly (dark blue) or our de novo BL6J assembly (lighter blue). Bigwigs from input, ChIP-seq and RNA-seq were generated with 1X genome coverage normalization. For the ChIP-seq experiment, a further normalization of ChIP/input is also shown. **d,** IGV snapshot of RNA-seq data over *Zfp990* gene showing how the read pileup of sequences on the 3'exon of the gene changes in comparison to the rest of the gene, when aligning the reads to the GRCm39 reference assembly (dark blue) or our de novo BL6J assembly (lighter blue). The large pileup comes from highly represented zinc finger sequences present in other KZFP genes throughout the Chr4 cluster.

Supplementary Fig. 3

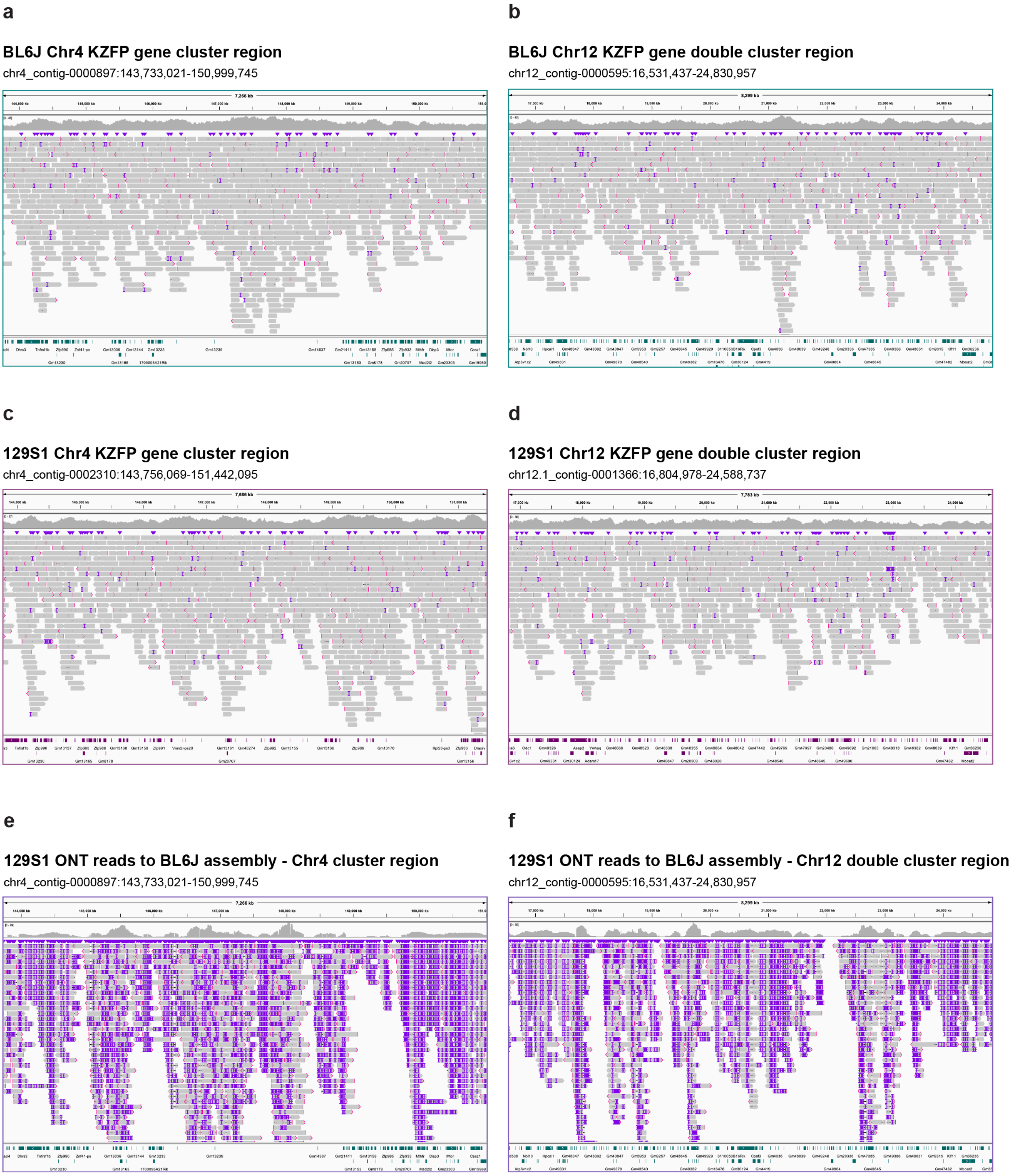

Supplementary Figure 3. Alignment of ultralong ONT reads to de novo assembled KZFP gene clusters.

a-b, BL6J haplotype ONT reads (>100kb) aligned to the BL6J assembly. IGV snapshots at genomic regions harboring the KZFP gene clusters on Chr4 (a) and Chr12 (b) demonstrate that the reads tile the assembly. Importantly, the de novo assembled Chr12 locus is identical to the region in the reference GRCm39 assembly (Supplementary Fig. 7b). **c-d,** 129S1 haplotype ONT reads (>100kb) aligned to the 129S1 assembly. IGV snapshots at genomic regions harboring the KZFP gene clusters on Chr4 (c) and Chr12 (d) demonstrate that the reads tile the assembly. **e-f,** 129S1 haplotype ONT reads (>100kb) aligned to the BL6J assembly (proof of principle alignment). As the reads are aligned

to the wrong assembly, the IGV snapshots at genomic regions harboring the KZFP gene clusters on Chr4 (e) and Chr12 (f) demonstrate that the reads do not continuously tile the loci, with some regions completely missing coverage while others present coverage spikes.

Supplementary Fig. 4

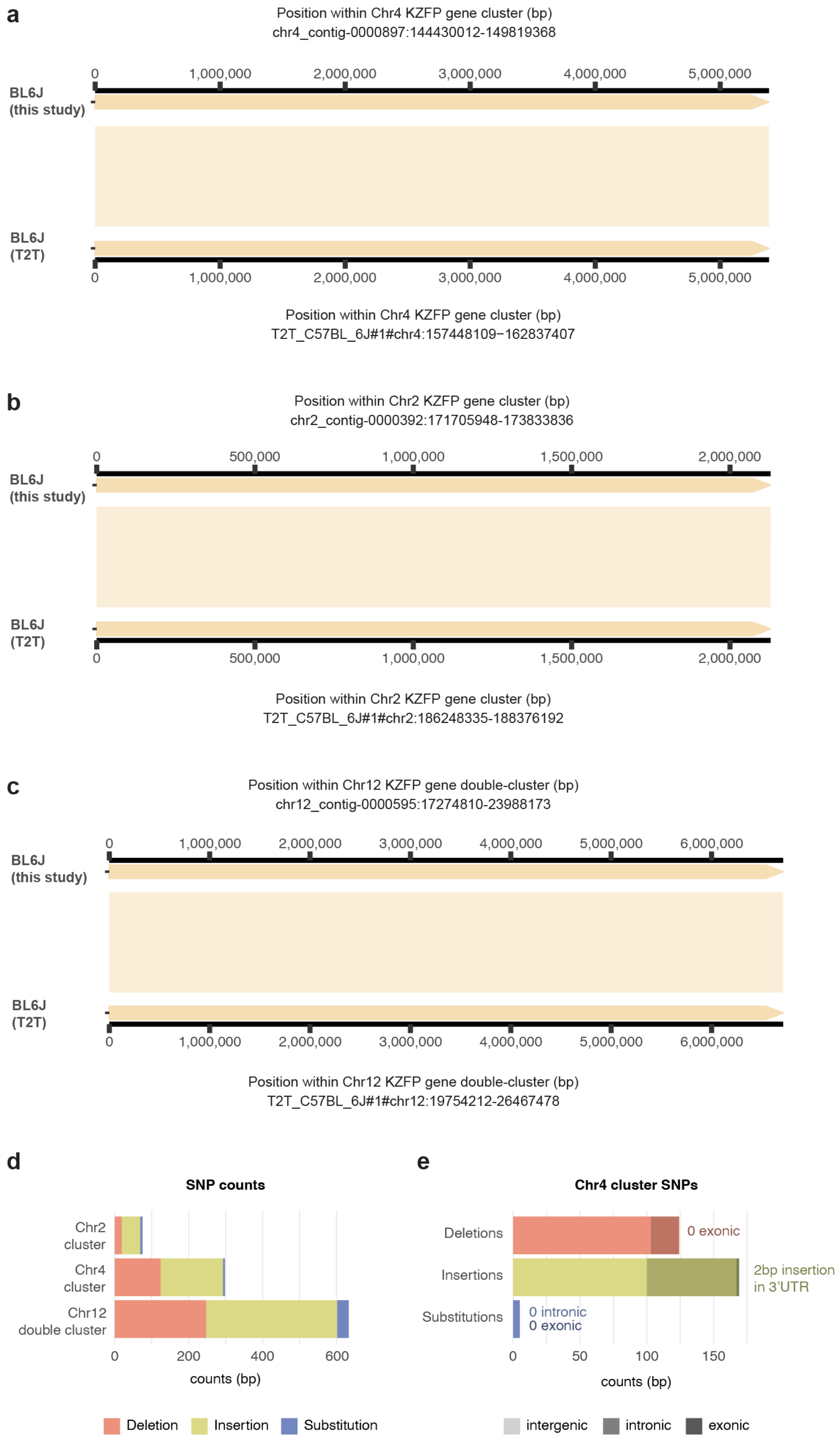

Supplementary Figure 4. Comparison of young KZFP gene clusters between two BL6J assemblies.

Alignment of our de novo assembly for the BL6J strain to the recently released C57BL/6J T2T assembly (GCA_964188535) from Francis, B., et al. (bioRxiv 2024) [26] at the Chr4 (**a**), Chr2 (**b**) and Chr12 (**c**) KZFP gene cluster loci. No structural variation between the assemblies was detected. (**d**) SNP counts (bp) at the KZFP clusters on Chr2, Chr4 and Chr12 detected by comparing the two BL6J. Different colors are used for different SNP types: red for deletions, green for insertions and blue for substitutions. (**e**) Detailed analysis of SNPs detected within the Chr4 KZFP gene cluster. Different color shades (from lightest to darkest) indicate whether SNPs occur in intergenic, intronic of exonic regions.

Supplementary Fig.5

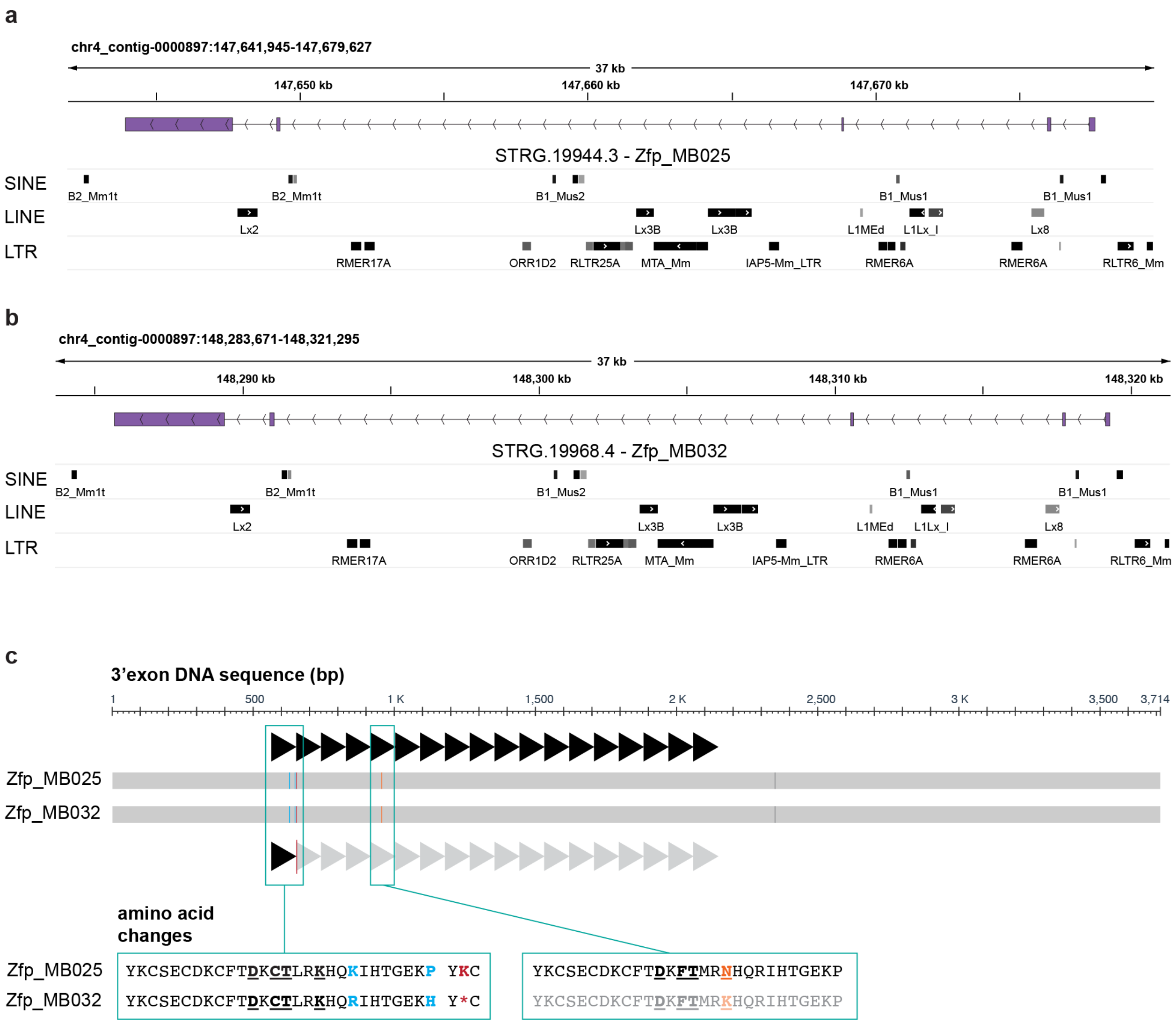

Supplementary Figure 5. Comparison of Zfp_MB025 and Zfp_MB032.
a-b, IGV snapshots of Zfp_MB025 (a) and Zfp_MB032 (b) gene annotations; the TE annotations highlight that the two genes are close copies. **c**, alignment of the whole 3' exon DNA sequence of Zfp_MB025 and Zfp_MB032 (top) and consequent amino acid differences (bottom). The black triangles indicate zinc finger domains encoded by the genes, while the grey triangles indicate the potential zinc fingers hidden after a premature stop codon in Zfp_MB032. Different color lines indicate the 4 SNPs that distinguish these two genes, including one leading to the premature stop codon in Zfp_MB032 (highlighted in red). Fingerprint amino acids are highlighted in bold and underline.

Supplementary Fig. 6

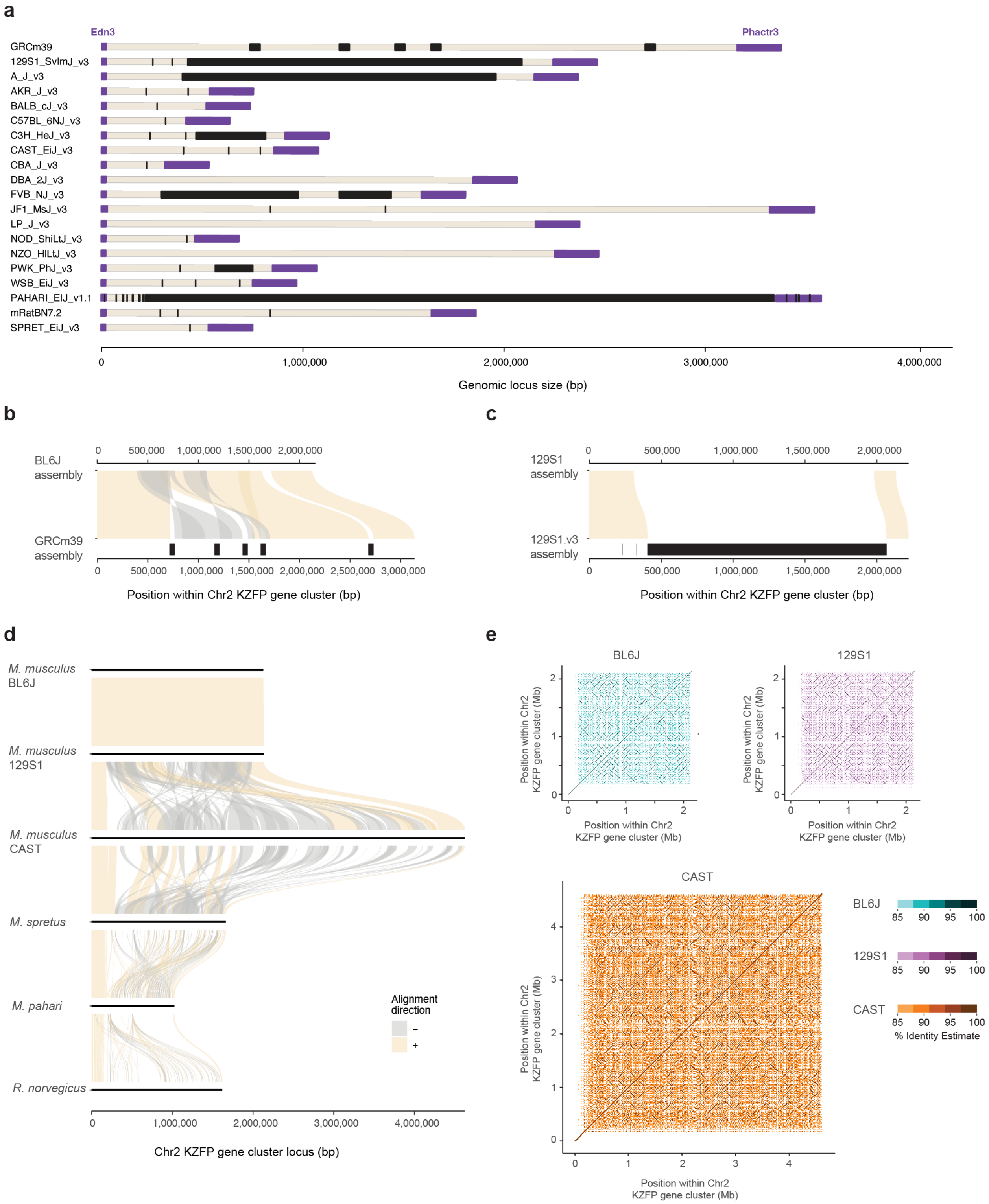

Supplementary Figure 6. Comparative analysis of the Chr2 KZFP gene cluster.
a. Annotation of sequence gaps (black rectangles) in the Chr2 KZFP gene cluster locus in different mouse strains and species, and rat. The genes *Edn3* and *Phactr3* (up- and downstream of the cluster, respectively) are shown in purple. **b**, Comparison of the Chr2 KZFP gene cluster (defined as 1bp downstream of the *Edn3* gene and 1bp upstream of the *Phactr3* gene) in our de novo BL6J assembly with the reference GRCm39 assembly. Gaps in the GRCm39 assembly are indicated in black. **c**, Comparison of the Chr2 KZFP gene cluster (defined as 1bp downstream of the *Edn3* gene and 1bp

upstream of the *Phactr3* gene) in our de novo 129S1 assembly with the available 129S1/SvImJ_v3 (GCA_921998555.2) assembly. Gaps in the 129S1.v3 assembly are indicated in black. **d,** Sequence comparison of the Chr2 KZFP gene cluster locus (defined as 1bp downstream of the *Edn3* gene and 1bp upstream of the *Phactr3* gene) between the three strains of *Mus musculus* with *Mus spretus*, *Mus pahari* and *Rattus norvegicus*.

e, Self-identity dotplots highlighting patterns of high sequence similarity within the Chr2 KZFP gene cluster in BL6J, 129S1 and CAST mouse strains.

Supplementary Fig. 7

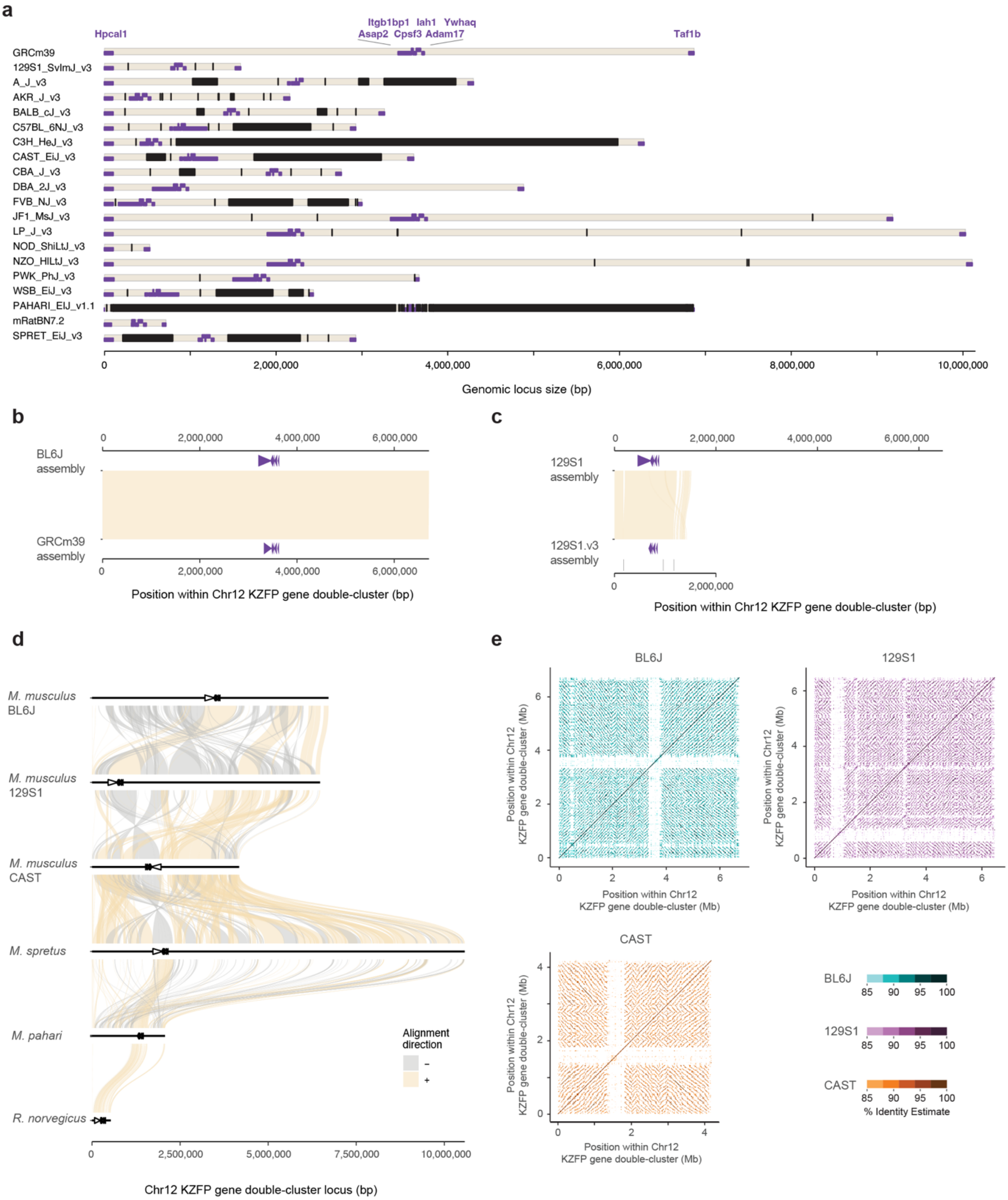

Supplementary Figure 7. Comparative analysis of the Chr12 KZFP gene double-cluster.
a. Annotation of sequence gaps (black rectangles) in the Chr12 KZFP gene double-cluster locus in different mouse strains and species, and rat. Non-KZFP genes upstream, downstream and between the two clusters are indicated in purple. **b**, Comparison of the Chr12 KZFP gene double-cluster (defined as 1bp downstream of the *Hpcal1* gene and 1bp upstream of the *Taf1b* gene) in our de novo BL6J assembly with the reference GRCm39 assembly. Non-KZFP genes between the two KZFP gene clusters are shown as purple arrow heads. **c**, Comparison of the Chr12 KZFP gene double-cluster (defined as 1bp downstream of the *Hpcal1* gene and 1bp upstream of the *Taf1b* gene) in our de novo

129S1 assembly with the available 129S1/SvImJ_v3 (GCA_921998555.2) assembly. Non-KZFP genes between the two KZFP gene clusters are shown as purple arrow heads. Very small gaps annotated in the 129S1.v3 assembly are indicated in black. **d,** Sequence comparison of the Chr12 KZFP gene double-cluster locus (defined as 1bp downstream of the *Hpcal1* gene and 1bp upstream of the *Taf1b* gene) between the three strains of *Mus musculus* with *Mus spretus*, *Mus pahari* and *Rattus norvegicus*. Non-KZFP genes between the two KZFP gene clusters are shown as arrow heads. **e,** Self-identity dotplots highlighting patterns of high sequence similarity within the Chr12 KZFP gene double-cluster in BL6J, 129S1 and CAST mouse strains.

Supplementary Fig. 8

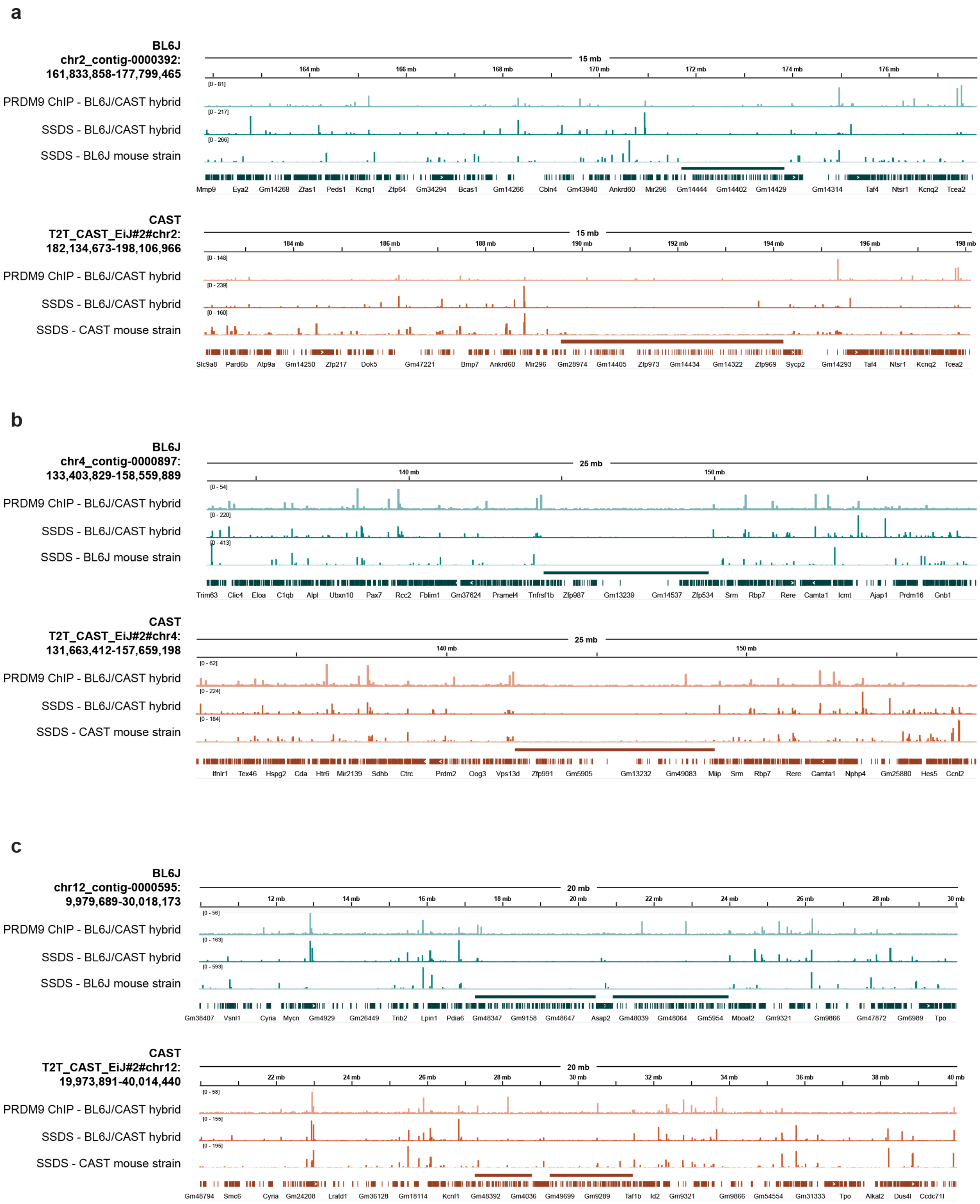

Supplementary Figure 8. Distribution of PRDM9 and of DMC1-bound single-stranded DNA at young KZFP gene clusters.

PRDM9 binding and DMC1-bound single-stranded DNA (SSDS) at the KZFP gene clusters on Chr2 (**a**), Chr4 (**b**) and Chr12 (**c**) and surrounding genomic regions in BL6J and CAST mouse strains. The position of the KZFP gene clusters is indicated as a line above the gene annotation track; liftover of the Gencode M32 gene annotation is shown as gene annotation track.

Supplementary Fig. 9

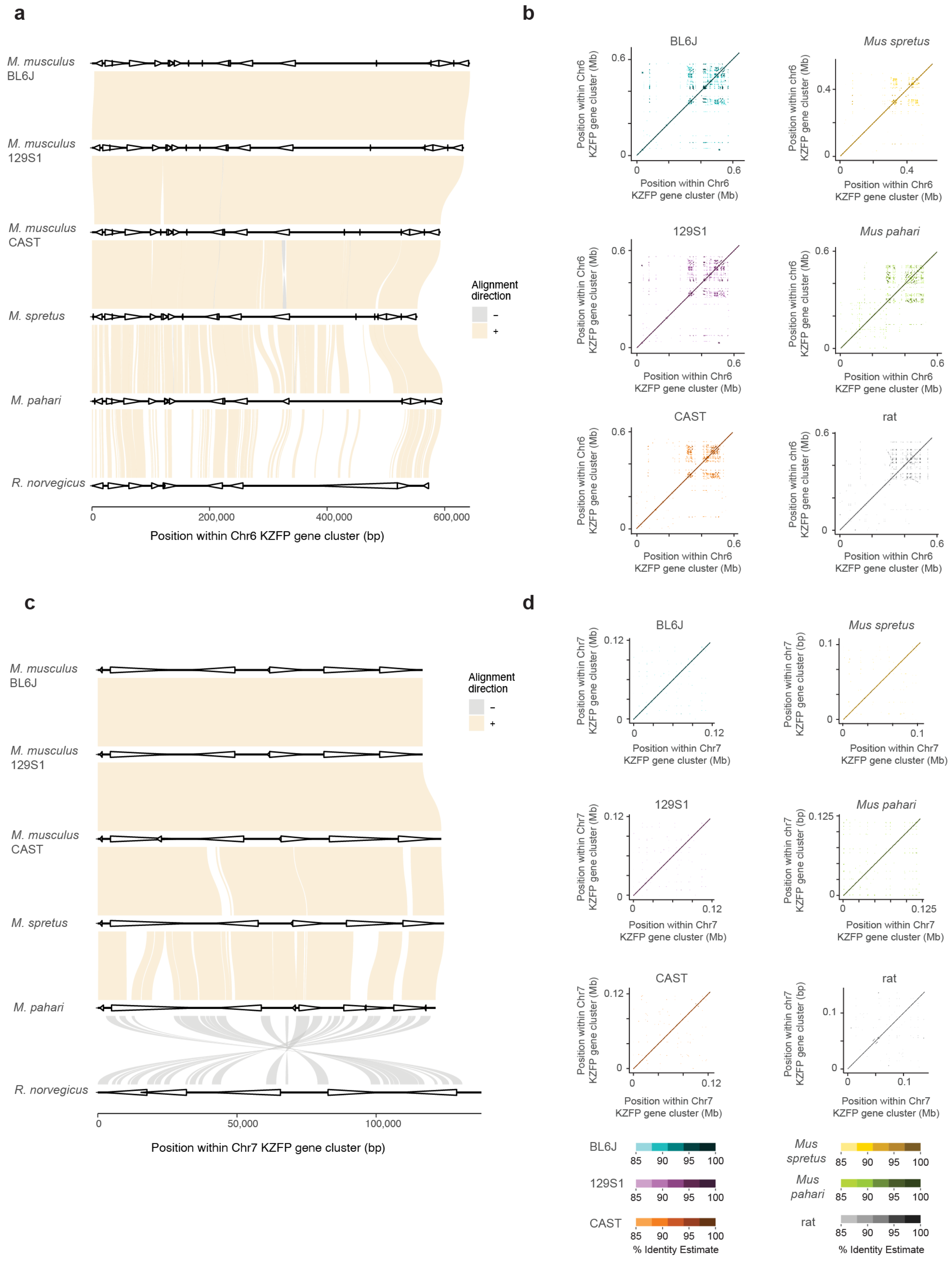

Supplementary Figure 9. Comparative analysis of old KZFP gene clusters on Chr6 and Chr7.

a, Sequence comparison of the Chr6 KZFP gene cluster locus between the three strains of *Mus musculus* with *Mus spretus*, *Mus pahari* and *Rattus norvegicus*. Genes in the cluster are shown as arrow heads.
b, Self-identity dotplots highlighting patterns of high sequence similarity within the Chr6 KZFP gene cluster in BL6J, 129S1 and CAST mouse strains. **c,** Sequence comparison of the Chr7 KZFP gene cluster locus between the three strains of *Mus musculus* with *Mus spretus*, *Mus pahari* and *nattus Norvegicus*. Genes in the cluster are shown as arrow heads. **d,** Self-identity dotplots highlighting patterns of high sequence similarity within the Chr7 KZFP gene cluster in BL6J, 129S1 and CAST mouse strains.

Supplementary Fig. 10

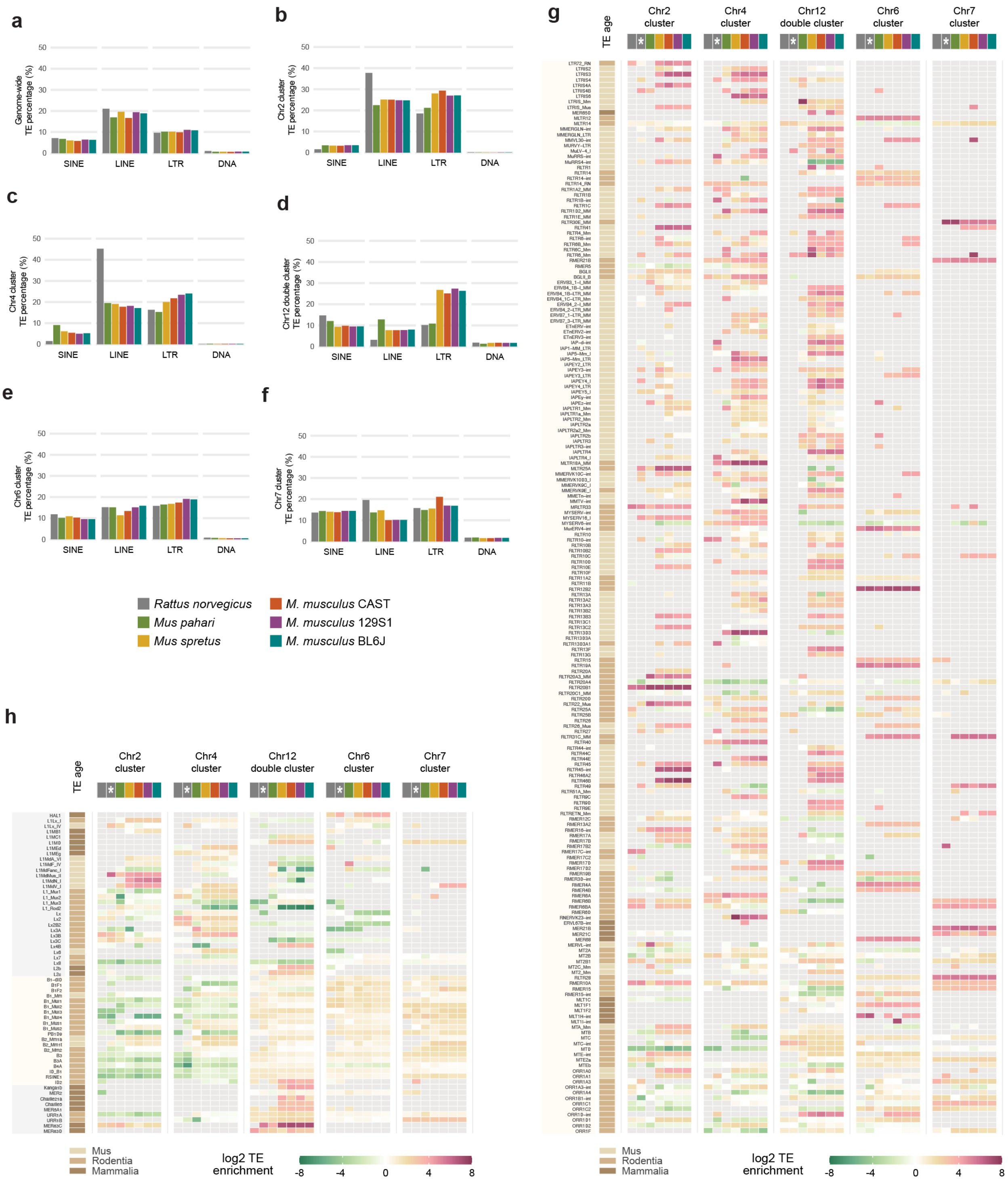

Supplementary Figure 10. TE representation at examined KZFP gene clusters.
a, Genome-wide TE representation by class in rat, *Mus pahari*, *Mus spretus* and three *Mus musculus* strains (BL6J, 129S1 and CAST). **b-f,** TE representation by class at KZFP gene cluster loci (from *Mus musculus* locations) at Chr2 (b), Chr4 (c), Chr12 (d), Chr6 (e) and Chr7 (f). **g-h,** Heatmap of enrichment of LTR (g) and LINE, SINE and DNA transposons (h) at the different KZFP gene cluster loci. Enrichment is shown as log2 of the ratio between the percentage of the KZFP gene cluster annotated as the ERV (bp) and the percentage of the whole genome annotated as the ERV (bp). Grey tiles indicate complete absence of the TE family in the corresponding locus. Species and mouse strains are indicated on top of each heatmap following the color lagend in (a) as follows: rat in grey,

Mus pahari in green, *Mus spretus* in yellow, CAST in orange, 129S1 in magenta and BL6J in teal. For rat, enrichment of TEs annotated in the rat RepeatMasker is shown, together with enrichment of TEs annotated using the mouse repeat library (column is highlighted with a white asterisk). TE conservation is indicated in brown shades (lightest shade indicates TEs only present in the Mus lineage, middle shade indicates Rodentia TEs and darkest shade indicates mammalian TEs).

Supplementary Fig. 11

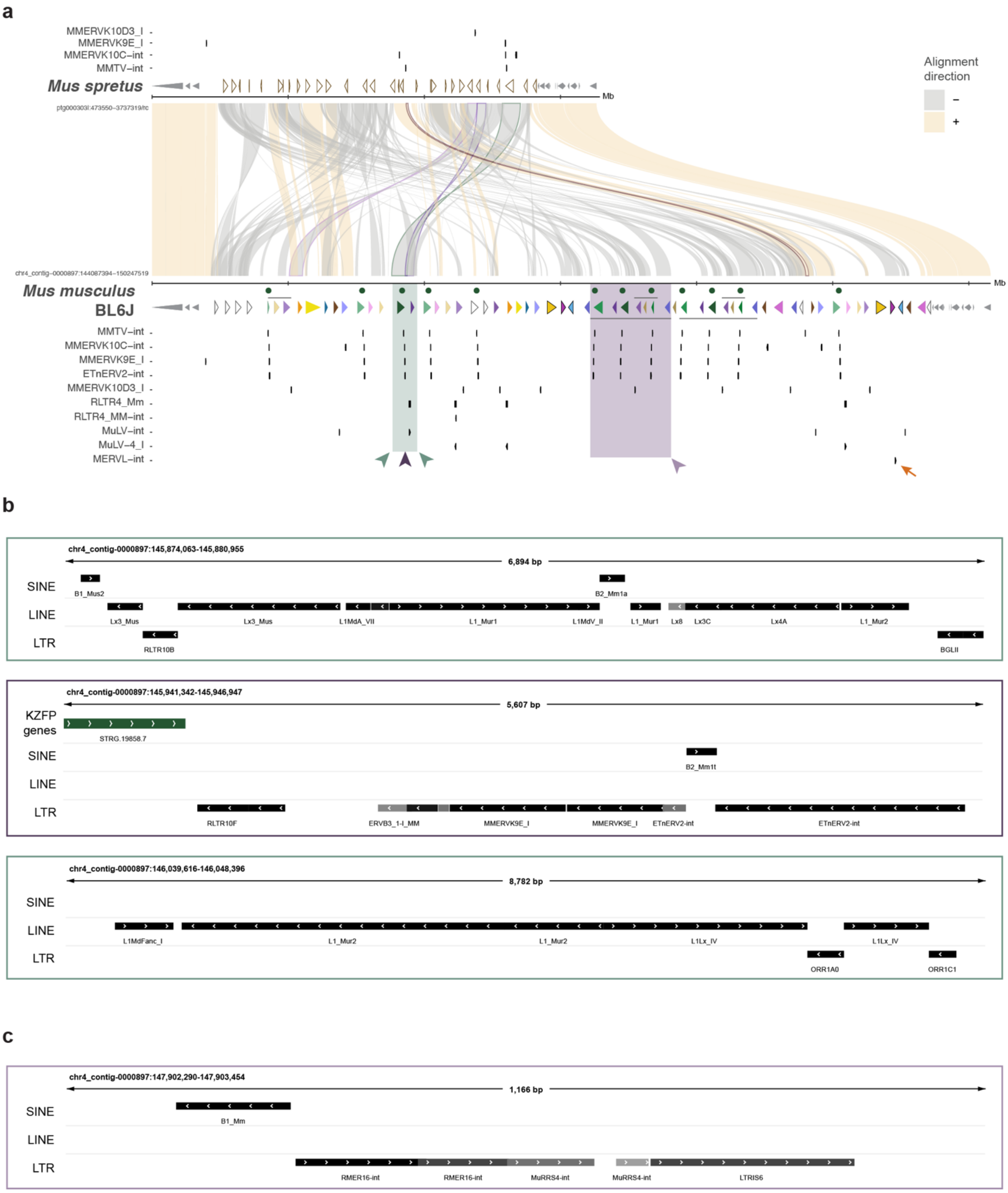

Supplementary Figure 11. Comparison of BL6J and *Mus spretus* Chr4 KZFP gene clusters highlights ERV duplications and ERV-mediated recombination events.
a, Comparison of the Chr4 KZFP gene cluster locus between *Mus spretus* and BL6J *Mus musculus*. BL6J KZFP genes are color coded as in Figure 4a for comparison, grey genes indicate non-KZFP genes outside of the cluster, while *Mus spretus* KZFP genes have been annotated based on liftoff of the BL6J KZFP gene annotation. ERV integrations of representative subfamilies are also displayed; no integrant of ETnERV2-int, RLTR4_Mm, RLTR4_MM-int, MuLV-int, MuLV-4_I and MERVL-int was found in the *Mus spretus* locus. The unique MERVL-int integration in the BL6J cluster is highlighted

by an orange arrow, like in Figure 4c. Alignments of two original MMTV-int integrations, conserved between *Mus spretus* and BL6J *Mus musculus*, are highlighted in brown (integrant that did not undergo duplication) and in dark green (integrant that underwent multiple duplication events in *Mus musculus*, highlighted by a dark green dot above the BL6J KZFP genes). Lines above and below BL6J KZFP genes highlight different orders of segmental duplication blocks, sharing identical patterns of MMTV-int, MMERVK10C-int, MMERVK9E_I, ETnERV2-int and MMERVK10D3_I integrations. Alignment of the initial conserved MMERVK10D3_I integration is highlighted in light purple, while alignment of the upstream portion of that locus is highlighted in dark purple. **b,** IGV snapshots of the recombination scars at the boundaries of the gene block highlighted by arrow heads at the green region in panel a (top and bottom LINE elements) and between the two KZFP genes in that block (middle chimeric ERV). ERV annotation was validated by unbiased search of the sequence in Dfam database, confirming fusion of the internal region of different ERV subfamilies with high sequence similarity. **c,** Chimeric ERV marking the recombination scar at the downstream boundary of the large duplicated block highlighted by the light purple arrowhead in panel a.

Supplementary Fig. 12

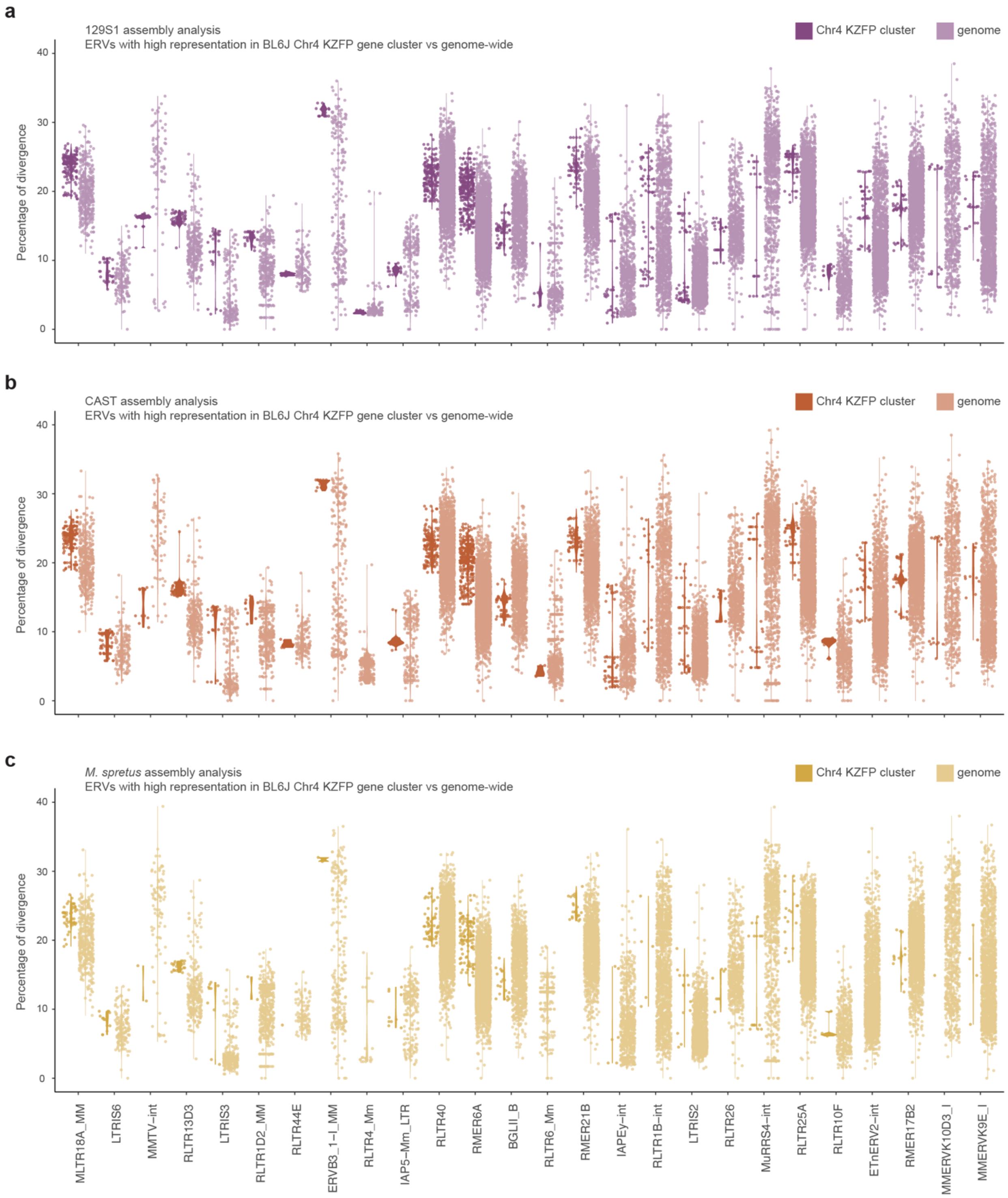

Supplementary Figure 12. Percentage of sequence divergence of 129S1, CAST and *Mus spretus* ERVs with high representation in the BL6J Chr4 KZFP gene cluster.
Percentage of divergence to consensus for ERVs from Fig. 5a, shown for the 129S1 (**a**) and CAST (**b**) strains, and for *Mus spretus* (**c**) at the Chr4 KZFP gene cluster locus (darker color) versus genome-wide (lighter color).

Supplementary Fig. 13

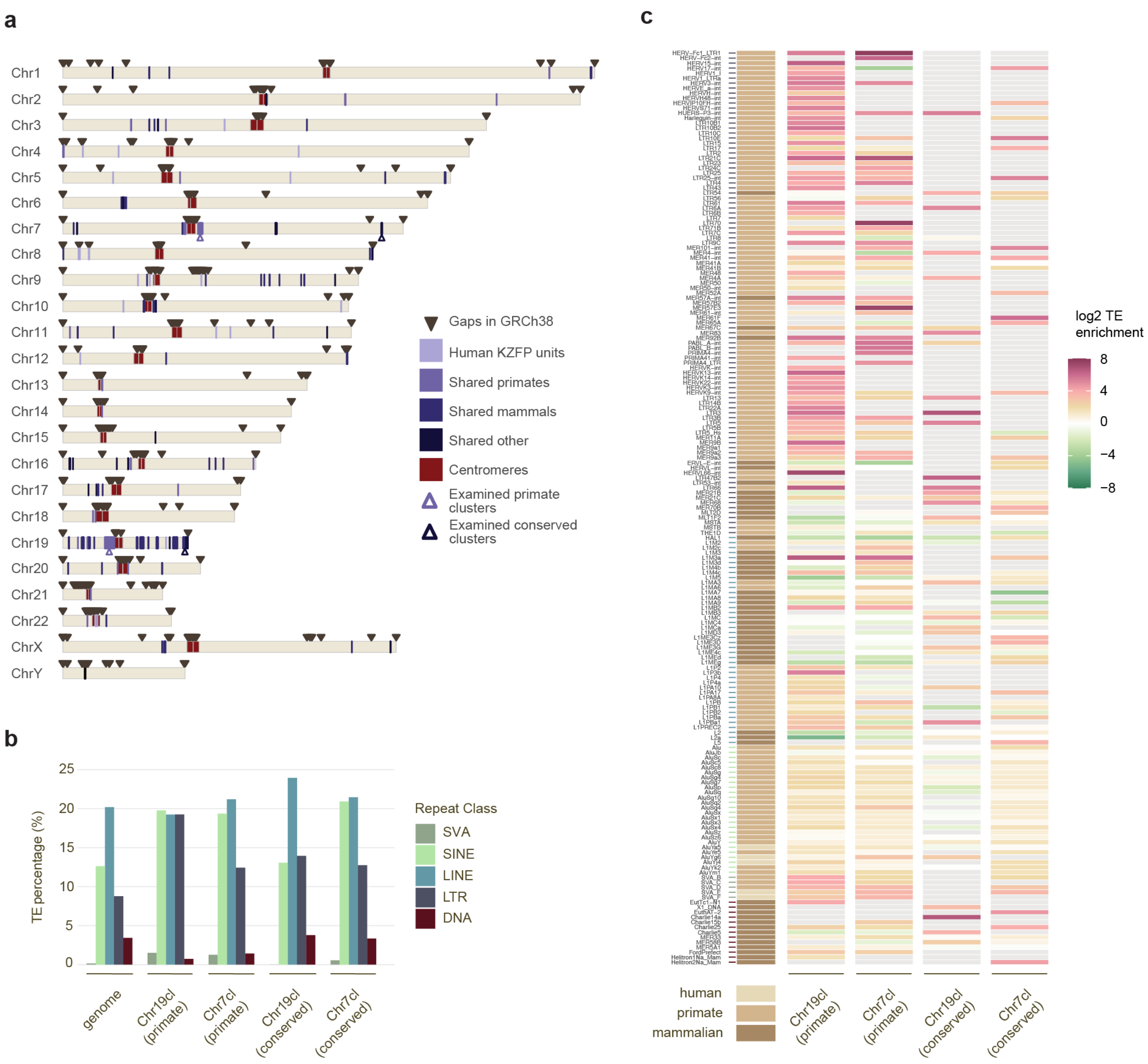

Supplementary Figure 13. KZFP gene cluster distribution and their TE composition in human. **a,** Genomic distribution in the human genome (GRCh38.p14 assembly) of KZFP units, colored by conservation. Assembly gaps are indicated by dark arrow heads above the locus. The position of the KZFP gene clusters examined in this study is highlighted with arrow heads below the loci. The primate cluster on Chr19 represents the largest KZFP gene cluster in the human genome, sized ~4.5Mb, and harboring ZNF91 and ZNF93 genes [53]. The conserved cluster at the end of human Chr19 corresponds to the mouse KZFP gene cluster on Chr7, while the conserved cluster at the end of human Chr7 corresponds to the mouse KZFP gene cluster on Chr6. **b,** TE representation by class genome-wide and at the examined KZFP gene cluster loci in the human genome. **c,** Heatmap of enrichment of TEs sorted by class and family at the different KZFP gene cluster loci in human. Enrichment is shown as log2 of the ratio between the percentage of the KZFP gene cluster annotated as the TE (bp) and the percentage of the whole genome annotated as the TE (bp). Grey tiles indicate complete absence of the TE family in the corresponding locus.
