# Supplementary material for: Young KRAB-zinc finger gene clusters are highly dynamic incubators of ERV-driven genetic heterogeneity in mice
Source: Nat Commun. 2025 Oct 30;16:9608. doi: 10.1038/s41467-025-64609-2 (PMC12575710; doi:10.1038/s41467-025-64609-2)
Supplement: Supplementary file 2 — Description of Additional Supplementary Files [file 41467_2025_64609_MOESM2_ESM.pdf]

Description of Additional Supplementary Files

Supplementary Data 1: Curated annotation of KZFP genes in the Chr4 cluster in BL6J, 129S1 and CAST mouse strains.

Supplementary Data 2: Zinc fingerprint arrays of coding KZFP genes in the Chr4 cluster in BL6J, 129S1 and CAST mouse strains.

Supplementary Data 3: TE content and enrichment at examined KZFP gene clusters in rat, *Mus pahari*, *Mus spretus*, *Mus musculus* (BL6J, 129S1 and CAST strains) and *Homo sapiens*.

Supplementary Data 4: Percentage of divergence of LTR elements in BL6J, 129S1 and CAST strains, and *Mus spretus*.

Supplementary Data 5: Enrichment analysis of BL6J Chr4cl KZFP ChIP-seq peaks over TEs.

Supplementary Data 6: Summary of BL6J Chr4cl KZFPs' binding preferences: target TEs and sequence motifs.
