# Supplementary material for: Young KRAB-zinc finger gene clusters are highly dynamic incubators of ERV-driven genetic heterogeneity in mice
Source: Nat Commun. 2025 Oct 30;16:9608. doi: 10.1038/s41467-025-64609-2 (PMC12575710; doi:10.1038/s41467-025-64609-2)
Supplement: Supplementary file 9 — Reporting Summary [file 41467_2025_64609_MOESM9_ESM.pdf]

nature portfolio

Corresponding author(s): Melania Bruno
Todd Macfarlan

Last updated by author(s): Sep 15, 2025

Reporting Summary

Nature Portfolio wishes to improve the reproducibility of the work that we publish. This form provides structure for consistency and transparency in reporting. For further information on Nature Portfolio policies, see our Editorial Policies and the Editorial Policy Checklist.

Statistics

For all statistical analyses, confirm that the following items are present in the figure legend, table legend, main text, or Methods section.

n/a	Confirmed	
[ ]	[x]	The exact sample size (*n*) for each experimental group/condition, given as a discrete number and unit of measurement
[x]	[ ]	A statement on whether measurements were taken from distinct samples or whether the same sample was measured repeatedly
[ ]	[x]	The statistical test(s) used AND whether they are one- or two-sided<br>*Only common tests should be described solely by name; describe more complex techniques in the Methods section.*
[x]	[ ]	A description of all covariates tested
[x]	[ ]	A description of any assumptions or corrections, such as tests of normality and adjustment for multiple comparisons
[ ]	[x]	A full description of the statistical parameters including central tendency (e.g. means) or other basic estimates (e.g. regression coefficient) AND variation (e.g. standard deviation) or associated estimates of uncertainty (e.g. confidence intervals)
[ ]	[x]	For null hypothesis testing, the test statistic (e.g. *F*, *t*, *r*) with confidence intervals, effect sizes, degrees of freedom and *P* value noted<br>*Give P values as exact values whenever suitable.*
[x]	[ ]	For Bayesian analysis, information on the choice of priors and Markov chain Monte Carlo settings
[x]	[ ]	For hierarchical and complex designs, identification of the appropriate level for tests and full reporting of outcomes
[x]	[ ]	Estimates of effect sizes (e.g. Cohen's *d*, Pearson's *r*), indicating how they were calculated

Our web collection on statistics for biologists contains articles on many of the points above.

Software and code

Policy information about availability of computer code

Data collection	PacBio circular consensus sequence (CCS)/HiFi reads were generated off-instrument from the initial subread data from each SMRTCell using the pb_ccs workflow (ccs version 6.3.0) within PacBio SMRTLink version 11.0.0.146107.<br>ONT sequencing was performed on ONT FLO-PRO002 and FLO-MIN106 flowcells; basecalling was done on instrument using Guppy v6.3.9, or Guppy v6.4.6 in high-accuracy mode.<br>PacBio Iso-Seq was performed on a Sequel IIe sequencer (Pacific Biosciences) running instrument control software version 11.0.0.144466 and a movie collection time of 25 hours per SMRTCell with 2hr pre-extension. CCS/HiFi reads were generated from the initial subread data using the pb_ccs workflow (ccs v.6.3.0) within PacBio SMRTLink version 11.0.0.146107.
Data analysis	Phylogenetic trees were generated using TimeTree and downloaded in Newick format.<br>For de novo assemblies, the following tools were used: Canu, Verkko and hifiasm.<br>BUSCO v5.4.7 was used to assess assembly completeness.<br>For sequence alignments the tools lastz and minimap2 were used.<br>MUMmer v 4.0.1 was used for alignment and SNP identification between BL6J assemblies.<br>For multiple sequence alignments the tools Clustal omega and MAFFT were used.<br>RepeatMasker v4.1.5 was used for repeat annotation.<br>Liftoff was used to lift known gene annotation from GRCm39 over other assemblies.<br>LIMA v.2.7.1 and IsoSeq3 v.3.8.2 were used to process and analyze PacBio Iso-Seq data.<br>STAR v.2.7.10b was used to align RNA-seq reads.<br>StringTie v.2.2.1 was used for de novo transcript assembly.<br>Integrative Genome Viewer and Snapgene softwares were used to navigate through data and for manual inspection of transcripts.

To analyze ChIP-seq data, the following tools were used: fastQC v0.12.1, BWA v0.7.17, SAMtools v1.19, deepTools v3.5.4, khmer v2.1.1, MACS2 v2.2.7.1, BEDTools v2.31.1, MEME suite v5.5.5.
The tools RCADE (Najafabadi et al, Bioinformatics, 2015) and the Zinc Finger Recognition Code (ZiFRC) (Najafabadi et al, Briefings in Bioinformatics, 2012) were used for KZFP target motif analysis.
Data tables analysis and plots were generated in RStudio, using the following R packages:
readr 2.1.5, dplyr 1.1.4, stringr 1.5.1, karyoploter 1.28.0, ggtree 3.10.1, svbyeye 0.99.0, phangorn 2.12.1 , ape 5.8, regioneR 1.34.0, ggplot2.

For manuscripts utilizing custom algorithms or software that are central to the research but not yet described in published literature, software must be made available to editors and reviewers. We strongly encourage code deposition in a community repository (e.g. GitHub). See the Nature Portfolio guidelines for submitting code & software for further information.

Data

Policy information about availability of data

All manuscripts must include a data availability statement. This statement should provide the following information, where applicable:

- Accession codes, unique identifiers, or web links for publicly available datasets
- A description of any restrictions on data availability
- For clinical datasets or third party data, please ensure that the statement adheres to our policy

All raw data generated in this study has been deposited in the SRA database under the BioProject PRJNA1219187 [https://www.ncbi.nlm.nih.gov/bioproject/?term=PRJNA1219187]. The ChIP-seq data generated in this study, together with the corresponding processed data files (normalized bigwig coverage files and peak files), has been deposited as a GEO series in the NCBI GEO database under accession number GSE292055 [https://www.ncbi.nlm.nih.gov/geo/query/acc.cgi?acc=GSE292055].
The Mus musculus C57BL/6J de novo assembly after contig filtering and strand correction generated in this study has been deposited at DDBJ/ENA/GenBank under the accession JBPRBH000000000; the version described in this paper is version JBPRBH010000000 [https://www.ncbi.nlm.nih.gov/nuccore/JBPRBH000000000] The Mus musculus 129S1/SvImJ de novo assembly after contig filtering and strand correction generated in this study has been deposited at DDBJ/ENA/GenBank under the accession JBPRBI000000000; the version described in this paper is version JBPRBI010000000 [https://www.ncbi.nlm.nih.gov/nuccore/JBPRBI000000000]. The Mus spretus SPR2 de novo assembly generated in this study has been deposited at DDBJ/ENA/GenBank under the accession JBQVYN000000000; The version described in this paper is version JBQVYN010000000 [https://www.ncbi.nlm.nih.gov/nuccore/JBQVYN000000000].
The raw data used to generate the Mus pahari assembly is available in the SRA database under the BioProject PRJNA966193 [https://www.ncbi.nlm.nih.gov/bioproject/?term=PRJNA966193].
The RNA-seq data used in this study are available in the SRA database under the BioProject PRJNA923323 [https://www.ncbi.nlm.nih.gov/bioproject/PRJNA923323]. Additional KZFP ChIP-seq data used in this study are available as a GEO series in the NCBI GEO database under accession number GSE115287 [https://www.ncbi.nlm.nih.gov/geo/query/acc.cgi?acc=GSE115287].
The T2T C57BL/6J assembly used in this study is available in the NCBI Genome database under the GenBank accession number GCA_964188535.1 [https://www.ncbi.nlm.nih.gov/datasets/genome/GCA_964188535.1/]. The T2T CAST/EiJ assembly used in this study is available in the NCBI Genome database under the GenBank accession number GCA_964188545.1 [https://www.ncbi.nlm.nih.gov/datasets/genome/GCA_964188545.1/].
The PRDM9 ChIP-seq data used in this study is available in the SRA database under accession number SRX689499 [https://www.ncbi.nlm.nih.gov/sra?term=SRX689499]. The DMC1 SSDS data used in this study is available in the GEO database as GEO series GSE75419 [https://www.ncbi.nlm.nih.gov/geo/query/acc.cgi?acc=GSE75419].

Research involving human participants, their data, or biological material

Policy information about studies with human participants or human data. See also policy information about sex, gender (identity/presentation), and sexual orientation and race, ethnicity and racism.

Reporting on sex and gender	Not applicable
Reporting on race, ethnicity, or other socially relevant groupings	Not applicable
Population characteristics	Not applicable
Recruitment	Not applicable
Ethics oversight	Not applicable

Note that full information on the approval of the study protocol must also be provided in the manuscript.

Field-specific reporting

Please select the one below that is the best fit for your research. If you are not sure, read the appropriate sections before making your selection.

☒ Life sciences ☐ Behavioural & social sciences ☐ Ecological, evolutionary & environmental sciences

For a reference copy of the document with all sections, see nature.com/documents/nr-reporting-summary-flat.pdf

Life sciences study design

All studies must disclose on these points even when the disclosure is negative.

Sample size	No statistical methods were used to predetermine sample size.
Data exclusions	No data was excluded from the analysis. Any filtering of processed data during analysis (contigs and ChIP-seq peaks) is explained in the methods section of the article and in the ChIP-seq section of this report.
Replication	PacBio HiFi and ONT whole genome sequencing were performed using multiple libraries generated from the same genomic DNA prep, to reach a minimum of 30x genome coverage. All other sequencing experiments were performed in one replicate, with the only exception of 4 independent replicates of negative control for ChIP-seq.
Randomization	No randomization was performed.
Blinding	The investigators were not blinded during data acquisition or analysis as this is not required for the experiments performed in this study.

Reporting for specific materials, systems and methods

We require information from authors about some types of materials, experimental systems and methods used in many studies. Here, indicate whether each material, system or method listed is relevant to your study. If you are not sure if a list item applies to your research, read the appropriate section before selecting a response.

Materials & experimental systems

n/a	Involved in the study	
☐	☒	Antibodies
☐	☒	Eukaryotic cell lines
☒	☐	Palaeontology and archaeology
☐	☒	Animals and other organisms
☒	☐	Clinical data
☒	☐	Dual use research of concern
☒	☐	Plants

Methods

n/a	Involved in the study	
☐	☒	ChIP-seq
☒	☐	Flow cytometry
☒	☐	MRI-based neuroimaging

Antibodies

Antibodies used	anti-H3K4me3 (Abcam ab8580)<br>anti-H3K9me3 (Abcam ab8898)<br>anti-HA tag (Abcam, ab9110)
Validation	Anti-H3K4me3 (Abcam ab8580) and anti-H3K9me3 (Abcam ab8898) are widely used antibodies to address these common histone marks by ChIP-seq and validation is shown on the manufacturer website. Anti-HA tag (Abcam, ab9110) is also a widely used antibody for ChIP-seq experiments and validation is shown on the manufacturer website.

Eukaryotic cell lines

Policy information about cell lines and Sex and Gender in Research

Cell line source(s)	BL6J/129S1 F1 hybrid mouse embryonic stem cells (XY) were blastocyst derived from a cross between a female BL6J and a male 129S1 mouse. Cells were tested for karyotype stability by mitotic chromosome spreading and counting.<br>BL6J/CAST F1 hybrid mouse embryonic stem cells (XY) were blastocyst derived from a cross between a male BL6J and a female CAST mouse, and described in Francis et al. 2025.<br>HGTC8 mESCs (XY) were obtained from Jun Cheng, who derived this cell line in the lab of Dr. Lisa Garrett (Cheng et al. 2004).<br>F9 Embryonic carcinoma cells were purchased from ATCC (CRL-1720).
Authentication	Cell lines used in this study were not authenticated.
Mycoplasma contamination	Cell lines were tested for mycoplasma contamination at the beginning of the project in a PCR-based assay and tested negative.
Commonly misidentified lines<br>(See ICLAC register)	No commonly misidentified cell lines were used in this study.

Animals and other research organisms

Policy information about studies involving animals; ARRIVE guidelines recommended for reporting animal research, and Sex and Gender in Research

Laboratory animals	The following mouse strains were used in this study: C57BL/6J (JAX #000664), 129S1/SvImJ (JAX #002448) and SPR2 Mus spretus (RIKEN RBRC00208). Adult mice (between 2 and 6 months of age) were used in this study. All mouse procedures had been reviewed and approved by the National Institute of Child Health and Human Development (NICHD) Animal Care and Use Committee (ACUC) at the National Institutes of Health (ASP#: 24-026).
Wild animals	No wild animal was used in this study.
Reporting on sex	Sex of the animals sequenced has been reported in the methods section of the study.
Field-collected samples	No field-collected sample was used in this study.
Ethics oversight	All animal studies were performed in accordance with NIH institutional guidelines.

Note that full information on the approval of the study protocol must also be provided in the manuscript.

Plants

Seed stocks	Not applicable
Novel plant genotypes	Not applicable
Authentication	Not applicable

ChIP-seq

Data deposition

- [x] Confirm that both raw and final processed data have been deposited in a public database such as GEO.
- [x] Confirm that you have deposited or provided access to graph files (e.g. BED files) for the called peaks.

Data access links<br>*May remain private before publication.*	https://www.ncbi.nlm.nih.gov/bioproject/?term=PRJNA1219187<br>https://www.ncbi.nlm.nih.gov/geo/query/acc.cgi?acc=GSE292055
Files in database submission	All raw sequencing data generated in this study has been deposited on SRA.<br>All processed data files for ChIP-seq experiments performed in this study (normalized bigwig and filtered peak files) have been deposited on GEO.
Genome browser session<br>(e.g. UCSC)	Normalized bigwigs of histone ChIP in C57BL/6J mESCs and of KZFP ChIP (ChIP/input) are accessible at the following link:<br>https://genome.ucsc.edu/s/melbruno/Bruno_et_al_KZFP

Methodology

Replicates	Experiments were performed in one replicate, except for negative control samples (4 independent samples)
Sequencing depth	Sample / number of reads<br>HGTC8_H3K4me3_ChIP 61590662<br>HGTC8_H3K9me3_ChIP 61812935<br>HGTC8_input 95924000<br>input_KZFP_ChIP_batch1 92092654<br>EV_control_rep1_KZFP_ChIP_batch1 63526220<br>Zfp978_ChIP_batch1 56946438<br>Zfp979_ChIP_batch1 43884017<br>Zfp982_ChIP_batch1 90444759<br>input_KZFP_ChIP_batch2 37852348<br>EV_control_rep2_KZFP_ChIP_batch2 40424371<br>Zfp6001_ChIP_batch2 31034661<br>Zfp6002_ChIP_batch2 32092423

Zfp6003_ChIP_batch2 44450315
input_KZFP_ChIP_batch3 30445204
Zfp981_ChIP_batch3 49082269
Zfp986_ChIP_batch3 44719463
Zfp988_ChIP_batch3 47509817
Zfp992_ChIP_batch3 76584651
Zfp993_ChIP_batch3 52659585
Zfp_MB003_ChIP_batch3 22816123
Zfp_MB004_ChIP_batch3 39452335
Zfp_MB005_ChIP_batch3 25412816
Zfp_MB010_ChIP_batch3 22929111
Zfp_MB011_ChIP_batch3 68495599
Zfp_MB012_ChIP_batch3 38165491
Zfp_MB013_ChIP_batch3 59566965
Zfp_MB014_ChIP_batch3 38480625
input_KZFP_ChIP_batch4 20902201
EV_control_rep3_KZFP_ChIP_batch4 52869454
EV_control_rep4_KZFP_ChIP_batch4 67796888
Gm21411_ChIP_batch4 25203004
Rex2_ChIP_batch4 19634387
Zfp268_ChIP_batch4 68998342
Zfp600_ChIP_batch4 23993481
Zfp980_ChIP_batch4 37893209
Zfp_MB001_ChIP_batch4 58477055
Zfp_MB015_ChIP_batch4 42348553
Zfp_MB016_ChIP_batch4 43051297
Zfp_MB018_ChIP_batch4 53836906
Zfp_MB020_ChIP_batch4 62624180
Zfp_MB021_ChIP_batch4 76878920
Zfp_MB022_ChIP_batch4 63811448
Zfp_MB023_ChIP_batch4 42550510
Zfp_MB024_ChIP_batch4 29451442
Zfp_MB025_ChIP_batch4 36466527
Zfp_MB026_ChIP_batch4 45969817
Zfp_MB027_ChIP_batch4 24908319
Zfp_MB028_ChIP_batch4 38070834
Zfp_MB029_ChIP_batch4 31392157
Zfp_MB031_ChIP_batch4 31098000
input_KZFP_ChIP_batch5 51575538
Zfp_MB035_ChIP_batch5 63196935
Zfp_MB036_ChIP_batch5 59142420
Zfp_MB037_ChIP_batch5 47633185

Antibodies

anti-H3K4me3 (Abcam ab8580)
anti-H3K9me3 (Abcam ab8898)
anti-HA tag (Abcam, ab9110)

Peak calling parameters

Reads were aligned to the Mus musculus GRCm39 reference genome assembly (GCF_000001635.27) for all the KZFP ChIP-seq experiments, using the Burrows-Wheeler Alignment (BWA) tool v0.7.17 (bwa aln and bwa sampe commands, default settings). Sam files were then converted into bam files with SAMtools v1.19, while removing eventually unmapped and duplicated reads, and retaining only primary alignments (samtools view -F 0x4,0x400,0x100,0x800 -b -h file.sam > file.bam). Bam files were sorted and indexed with SAMtools and converted to bigwig normalized to 1x genome coverage (RPGC normalization) for each sample with deepTools v3.5.4 (bamCoverage --bam file.bam -o file.bw -of bigwig --binSize 10 --effectiveGenomeSize 2521902382 --normalizeUsing RPGC --extendReads 200). ChIP bigwigs were further normalized by the input of the respective batch using deepTools (bigwigCompare -b1 ChIP.bw -b2 input.bw -o ChIP_input_ratio.bw -of bigwig --operation ratio –skipZeroOverZero --binSize 10).
The same analysis strategy was used also to analyze the H3K4me3 and H3K9me3 ChIP-seq data from HGTC8 cells. To generate bigwig normalized to 1x genome coverage for reads aligned to the de novo BL6J or CAST assembly, we used --effectiveGenomeSize 2525297504 or 2630722264, respectively, calculated using the unique-kmers.py command of the tool khmer v2.1.1 (with -k 200).
Peaks from the KZFP ChIP-seq experiments were called using MACS2 v2.2.7.1 (macs2 callpeak -t ChIP.bam -c input.bam -f BAMPE -g 2521902382). Peaks with even 1bp overlap with peaks called in any of the negative control replicate samples were removed.
Peaks were further filtered to only retain the ones with qValue =< 0.01 and fold enrichment over input of 10. For the samples that retained less than 20 peaks, fold enrichment over input of 5 was used as cutoff.

Data quality

Read quality was assessed with the FastQC tool.

Software

To analyze ChIP-seq data, the following tools were used: fastQC v0.12.1, BWA v0.7.17, SAMtools v1.19, deepTools v3.5.4, khmer v2.1.1, MACS2 v2.2.7.1, BEDTools v2.31.1, MEME suite v5.5.5.
